# Determinants of vaccination coverage and adherence to the Greek national immunization program among infants aged 2-24 months at the beginning of the economic crisis (2009-2011)

**DOI:** 10.1186/1471-2458-14-1192

**Published:** 2014-11-20

**Authors:** Papaevangelou Vassiliki, Koutsoumbari Ioanna, Vintila Artemis, Klinaki Eleni, Zellos Aglaia, Achilleas Attilakos, Tsolia Maria, Kafetzis Dimitris

**Affiliations:** Third Pediatric Department, National and Kapodistrian University of Athens Medical School, University General Hospital “ATTIKON”, Rimini 1, Chaidari, 12462 Greece; Second Pediatric Department, National and Kapodistrian University of Athens Medical School, “P&A Kyriakou” Childrens Hospital, Thivon & Livadias, Goudi, 11527 Greece; Hellenic Health Foundation, Athens, Greece; First Pediatric Department, National and Kapodistrian University of Athens Medical School, “Agia Sophia” Childrens Hospital, Thivon & Livadias, Goudi, 11527 Greece

**Keywords:** Vaccination, Coverage, Immigrants, Economic crisis, Socio economic factors

## Abstract

**Background:**

Childhood immunization has significantly reduced the incidence of vaccine preventable diseases. Parental mistrust over vaccine safety has been associated with vaccine refusal creating barriers on vaccine coverage. Recently, economic crisis has imposed additional impediment.

**Methods:**

Study aim was to evaluate vaccine coverage among infants 2-24 months old in the Athens metropolitan area at the beginning of the economic crisis (2009-2011).

**Results:**

Overall, 1,667 infants were enrolled (mean age 13 months). Less than 5% of parents admitted omitting or postponing vaccination secondary to their beliefs. Although vaccination coverage was acceptable for most vaccines, lower rates of immunization were found for some newer vaccines such as hepatitis A and rotavirus. Multiple regression analysis indicated that parental age, occupational, educational statuses and family size were independently associated with immunization coverage at 6 and 12 months. Interestingly, lack of insurance was not associated with missed vaccine doses.

**Conclusion:**

Incomplete vaccination coverage was associated with socioeconomic factors. It becomes apparent, that reassessing vaccination priorities under the current economic situation may be needed.

## Background

Vaccination is one of the most cost-effective public health initiatives that prevents up to 24% of the 10-12 million of yearly deaths of children under the age of 5 years [1]. The Expanded Program of Immunization (EPI) was initially adopted by World Health Organization (WHO) in 1974 to ensure that infants are fully immunized with the recommended childhood vaccines. Individual governments implement their own policies for vaccination programs following the international guidelines set by WHO.

In Greece, the National Immunization Program (NIP) is set by the Ministry of Public Health and Social Solidarity and is provided free of charge to all residents including immigrants (Table 1) [2]. At the individual level, adherence to the NIP offers protection against infectious diseases decreasing related morbidity and mortality [3]. In addition, high vaccination coverage at population level ensures development of herd immunity against infectious diseases and protects unimmunized susceptible individuals (too young to get vaccinated, immunocompromised, etc) [4]. Recently, parental concerns about vaccine safety together with the growing complexity of the immunization schedules and the increasing number of injections has lead to a rising number of parents who elect to postpone or even refuse the administration of some vaccines for their children [5]. This attitude poses their children at risk of acquiring a vaccine preventable disease (VPD) [6, 7]. Moreover, in close societies where many parents elect to postpone selected vaccines, risk of loss of herd immunity and reemergence of VPDs may occur increasing the chance of outbreaks [6, 7]. Additionally, in Greece, recent financial crisis has had a significant impact on access to healthcare services possibly severely affecting vaccination coverage [8, 9]. Therefore, in order to measure adherence to the NIP an ongoing surveillance system evaluating vaccination coverage, along with other measures including education of the public are necessary. Such systems have been implemented in many countries [10]. We report the results of a prospective survey conducted in 2009-2011, at the beginning of the economic crisis, to assess the vaccination coverage and timely adherence to vaccination scheme among Greek infants aged 2-24 months residing in Metropolitan Athens.

**Table 1:** **Greek National Immunization Programme (NIP) for children (2011)**

	Birth	2 months	4 months	6 months	12 months	15 months	18 months	24 months	4-6 years	11-12 years
HepB^1^		HepB	HepB	HepB						
DTaP^2^		DTaP	DTaP	DTaP		DTaP			DTap	
IPV^3^		IPV	IPV	IPV					IPV	
Hib^4^		Hib	Hib	Hib	Hib					
MCC^5^		MCC	MCC		MCC					
PCV^6^		PCV	PCV	PCV	PCV					
MMR^7^					MMR				MMR	
Varicella					Var				Var	
HepA^8^					Hep A (2 doses)					
TB^9^	BCG^10^								BCG^10^	

^1^HepB = hepatitis B vaccination. The recommended schedule for infants of HBsAg (-) is shown. However, for infants born to HBsAg(+) women, passive and active immunoprophylaxis is provided at birth and two additional vaccine doses at the ages of 1-2 months and 6-18 months respectively.

^2^DTaP = diphteria, tetanus and acellular pertussis vaccine.

^3^IPV = inactivated polio vaccine.

^4^Hib = conjugated haemophilus influenzae type B vaccine.

^5^MCC = meningococcal serotype C conjugated vaccine.

^6^PCV = pneumococcal conjugated vaccine.

^7^MMR = measles, mumps and rubella vaccine.

^8^HepA = hepatitis A vaccine.

^9^TB = tuberculosis.

^10^BCG = Bacillus Calmette–Guérin vaccine. Has been recommended to high risk children at birth since 2011.

## Methods

### Target population

This study evaluated vaccination coverage rates of infants visiting a tertiary public outpatient care clinic for either acute or routine health visit during 2009-2011. Study was approved form the “P & A Kyriakou Children’s” Hospital Ethics committee. All infants, aged 2-24 months, whose parents and/or legal representative were permanent residents of the greater urban district area of Attica, Greece were informed about the study. Their children were enrolled after informed written consent was provided. Children with underlying chronic disease such as immunodeficiency, extreme prematurity, neurologic diseases or other medical conditions interfering with vaccination administration where excluded. In order to identify predictors of incomplete immunization coverage, parents and/or legal representative of the infant were asked to complete a brief questionnaire to obtain information on demographic and socioeconomic characteristics, health care providers and utilization of immunization health services. The infant was considered as immunized or not based on the immunization card, as provided by the National Greek Health Care System. Parents who did not bring their child’s immunization card to the study visits were included in the study and analyzed as a subgroup.

The vaccination coverage for each vaccine was noted. Infants were considered as “adequately immunized” if they had received all vaccines, according to the Greek NIP (Table 1) with ≤2 months delay. To evaluate compliance with the vaccination scheme, 6 month old infants were considered “adequately immunized” if they had received 2 doses of diphtheria, tetanus toxoid and pertussis vaccine (DTP), 2 doses of poliovirus vaccine, 2 doses of *Haemophilus influenzae* type b vaccine (Hib), 1 dose of conjugated pneumococcal vaccine (PCV) and 1 dose of conjugated vaccine against meningococcus C. Respectively, at the age of 12 months they were considered “adequately immunized” if they had received 3 doses of diphtheria, tetanus toxoid and pertussis vaccine (DTP), 3 doses of poliovirus vaccine, 3 doses of *Haemophilus influenzae* type b vaccine (Hib), 2 doses of conjugated pneumococcal vaccine (PCV) and 1 dose of conjugated vaccine against meningococcus C. The decision for these definitions was based on common practices adopted by Greek pediatricians as discussed below. Moreover, rotavirus vaccination was not included since the vaccine was introduced in the NIP in 2011.

### Statistical analysis

Descriptive statistics are summarized as means ± standard deviations (SD) for the continuous variables and as frequencies (N) and percentages (%) for the categorical variables. Univariate logistic analysis was used to assess the association of vaccination coverage with each covariate in isolation.

Multiple logistic regression models are employed with a stepwise variable selection procedure examining those factors found significant during univariate analysis to identify significant variables affecting vaccination coverage. The chosen significant effects were further simplified using multiple comparison methods. All tests were performed at 5% level of significance. Statistical results were derived using the statistical package R (version 3.0.2) [http://CRAN.R-project.org/doc].

## Results

### Characteristics of study population

Overall, 1,667 infants were enrolled during the study period with a mean age 13.1 ± 6.6 months (51.5% boys). Enrollment was offered in 1,837 infants (response rate 90.7%). Main reason given for refusal was lack of time. Demographic and socioeconomic characteristics of the children and their parents as well as patterns of using medical care are shown in Table 2.

**Table 2:** **Demographic and socioeconomic characteristics of the study population**

Characteristics	N (%)
**Nationality**	**Total N = 1659**
Non-minority children	817 (49.25)
Minority (immigrants, Roma)	842 (50.75)
**Place of residence**	**Total N = 1192**
Inner city Athens	1068 (89.6)
Suburbs	124 (10.4)
**Child’s health insurance**	**Total N = 1647**
Public	1047 (63.57)
Social welfare insurance	130 (7.89)
Uninsured	131 (7.95)
Other (private, etc)	339 (20.58)
**Mother’s age**	**Total N = 1665**
≤20	133 (7.99)
21-30	785 (47.15)
31-40	687 (41.26)
≥41	60 (3.6)
**Father’s age**	**Total N = 1649**
≤20	66 (4.00)
21-30	457 (27.71)
31-40	845 (51.24)
≥41	281 (17.04)
**Mother’s education**	**Total N =1648**
Illiterate	159 (9.65)
< high school	281 (17.05)
High school	750 (45.51)
>high school	458 (27.79)
**Father’s education**	**Total N =1621**
Illiterate	364 (22.46)
< high school	143 (8.82)
High school	271 (16.72)
>high school	843 (52.00)
**Mother’s occupation**	**Total N = 1664**
Unemployed	1052 (63.22)
Worker	22 (1.32)
Public sector employee	123 (7.39)
Private sector employee	467 (28.06)
**Father’s occupation**	**Total N = 1628**
Unemployed	268 (16.46)
Worker	348 (21.38)
Public sector employee	166 (10.20)
Private sector employee	846 (51.96)
**Where do you go when your child is sick?**	**Total N =C1642**
Private Doctor	983 (59.87)
Hospital emergency room	433 (26.37)
Social Security outpatient services	183 (11.14)
Health Center	43 (2.62)
**Regular pediatrician?**	**Total N = 1657**
Yes	1194 (27.77)
No	463 (71.63)
**Where do you go to get your child vaccinated?**	**Total N = 1586**
Private paediatrician	866 (54.6)
Social Security outpatient services	481 (30.32)
Public outpatient clinic	239 (15.07)
**Anthropometric measurements over last 24 months**	**Total N = 1361**
0 – 3 times	236 (17.34)
> 4 times	1125 (82.66)

Overall, 180 children (10.8%) were brought without bringing their child's immunization card along. Importantly this group of parents were more likely to have more than 3 children (16.2%, 95% CI 11.9%-20.6%, p < 0.01), had finished at least high school (for mothers 11.8%, 95% CI 10.0%-13.7%, p < 0.01, for fathers 12.5%, 95%CI 10.6%-14.4% p < 0.01), Greek nationality (12.7%, 95% CI 10.4%-15.0%, p = 0.02) and reported visiting the emergency room (ER) for sick visits (14.1%, 95% CI 10.8%-17.4%, p < 0.01). Roma children constituted 9.8% of our population, of which, 63.8% visited the hospital without their immunization card.

Most parents were able to recall at least one postponement of vaccination (57%, 95% CI 54.6%-59.6%). Child's suffering from infection was the most frequent reason identified (37.1%, 95% CI 34.0%-40.2%) while less often parents had forgotten (9.3%, 95% CI 34.0%-40.2%) or where living too far from a vaccination center (5.1%, 95% CI 3.7%-6.6%). Finally, 79 parents (4.9%, 95% CI 3.9%-6.0%) admitted omitting a vaccine due to their belief that this vaccine was either not necessary or potentially harmful. Parents with no insurance (6.4%) did not report higher rates of missing vaccination (57.8%, 95% CI 55.1%-60.7%).

### Overall vaccination coverage in the study population

Immunisation coverage for each vaccine is shown in Table 3. Vaccination was confirmed by documentary evidence (immunization card). Coverage was highest for DTP-IPV-Hib. Among the more recently introduced vaccines, children had high vaccination coverage for varicella while coverage for hepatitis A and especially rotavirus vaccine was low (Table 3).

**Table 3:** **Immunization coverage in our study population aged 2-24 months**

Vaccine	n/N(%)	95% CI
**DTaP-IPV-Hib**		
≥ 2 doses at 6 months	1.147/1.297 (88.4%)	86.7% - 90.2%
≥ 3 doses at 12 months	891/1.008 (88.4%)	86.4% - 90.4%
**PCV**		
≥ 1 dose at 6 months	1.012/1.325 (77.1%)	74.8% - 79.3%
≥ 2 doses at 12 months	785/995 (78.9%)	76.4% - 81.4%
**MCC**		
≥ 1 dose at 6 months	518/1.279 (40.5%)	37.8% - 43.2%
**HepB**		
≥ 1 dose at 6 months	934/1.353 (69.0%)	66.6% - 71.5%
≥ 3 doses at 12 months	575/746 (77.1%)	74.1% - 80.1%
MMR*	287/356 (80.6%)	76.3% - 84.1%
Varicella*	308/352 (87.5%)	83.8% - 91.2%
Rotavirus (2 doses)	152/1.475 (10.3%)	8.8% - 11.9%
**HAV** (one dose at 24 months)	64/151 (42.4%)	34.5% - 50.3%

*Since according to NIP MMR and varicella should be administered at 12-15 months, children ≥17 months were evaluated.

DTaP = diphteria, tetanus and acellular pertussis vaccine.

IPV = inactivated polio vaccine.

Hib = conjugated haemophilus influenzae type B vaccine.

MCC = meningococcal serotype C conjugated vaccine.

HepB = hepatitis B vaccination.

MMR = measles, mumps and rubella vaccine.

HAV = hepatitis A vaccine.

### Factors associated with immunization coverage

**Figure 1:**
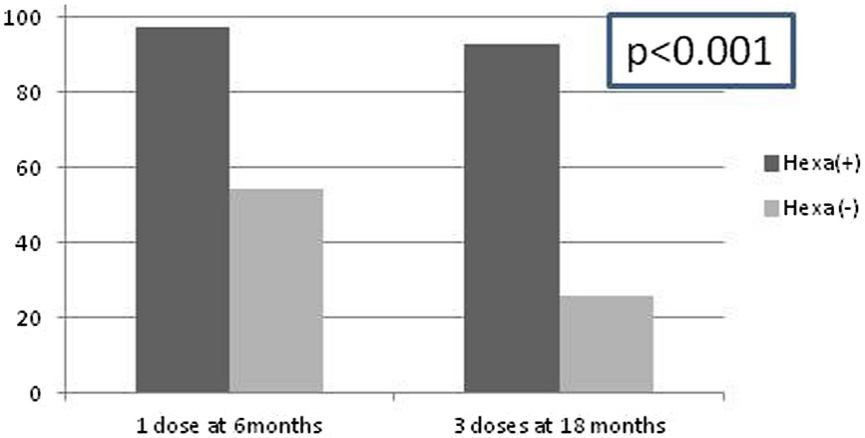
**Use of hexavalent vaccine was strongly associated with timely hepatitis B vaccination.**

Infants were less likely to be fully vaccinated if they belonged to a minority group, had more than one sibling, had young (<20 years old) or illiterate parents, had other than public insurance or their parents were unemployed (Table 4). Interestingly, appropriate immunization against hepatitis B (≥1 and 3 doses at 6 and 12 months respectively) was strongly associated with the use of the hexavalent vaccine (97.5% versus 54.2% and 92.8% versus 25.7% respectively, p < 0.01, Figure 1). Of note, although children belonging to minority groups (immigrants, Roma) were less likely to have received the hexavalent vaccine (15.6% versus 32,4%, p < 0.01), their vaccination coverage rates against hepatitis B were comparable to those of Greek children (73,2% versus 80,9%, p = 0.16).

**Table 4:** **Socioeconomic characteristics associated with full immunization**

	6 months			12 months		
Characteristics	n/N (%)	RR	95%CI	n/N (%)	RR	95%CI
**Minority**						
No	593/627 (94.6%)			481/513 (93.8%)		
Yes	546/662 (82.5%)	0.87	0.84-0.91	402/487 (82.5%)	0.88	0.84-0.92
**Family composition**						
One child	533/589 (90.5%)			436/478 (91.2%)		
Two children	474/532 (89.1%)	0.98	0.95-1.02	351/400 (87.8%)	0.96	0.92-1.01
More than 3 children	138/174 (79.3%)	0.88	0.81-0.95	102/128 (79.7%)	0.87	0.80-0.96
**Child’s health insurance**						
Public	733/793 (92.4%)			583/645 (90.4%)		
Other	397/486 (81.7%)	0.88	0.84 - 0.93	295/348 (84.8%)	0.94	0.89-0.99
**Mother’s age**						
≤20	43/89 (48.3%)			26/54 (48.1%)		
21-30	552/613 (90.0%)	1.86	1.50-2.31	436/495 (88.1%)	1.83	1.38-2.42
31-40	500/541 (92.4%)	1.91	1.54-2.37	385 / 415 (92.8%)	1.93	1.46-2.54
≥41	50/52 (96.2%)	1.99	1.59-2.48	42 / 42 (100.0%)	NA	NA-NA
**Father’s age**						
≤20	23/46 (50.0%)			11/26 (42.3%)		
21-30	284/350 (81.1%)	1.62	1.21-2.18	231/279 (82.8%)	1.96	1.25-3.08
31-40	626/661 (94.7%)	1.89	1.42-2.53	472/515 (91.7%)	2.17	1.38-3.40
≥41	205/229 (89.5%)	1.79	1.34-2.40	170/181 (93.9%)	2.22	1.41-3.48
**Mother’s education**						
Illiterate	57/111 (51.4%)			28/71 (39.4%)		
< high school	180/225 (80.0%)	1.56	1.29-1.89	132/163 (81.0%)	2.05	1.52-2.77
High school	538/567 (94.9%)	1.85	1.54-2.22	425/450 (94.4%)	2.39	1.79-3.20
>high school	355/377 (94.2%)	1.83	1.53-2.20	29/307 (94.5%)	2.40	1.79-3.20
**Father’s education**						
Illiterate	49/102 (48.0%)			29/66 (43.9%)		
< high school	180/219 (82.2%)	1.71	1.39-2.11	119/154 (77.3%)	1.76	1.32-2.34
High school	609/646 (94.3%)	1.96	1.60-2.40	499/529 (94.3%)	2.15	1.63-2.82
>high school	276/292 (94.5%)	1.97	1.60-2.41	214/228 (93.9%)	2.14	1.62-2.81
**Mother’s occupation**						
Unemployed	422/519 (81.3%)			332/406 (81.8%)		
Blue collar Worker	11/15 (73.3%)	0.90	0.66-1.23	8/11 (72.7%)	0.89	0.62-1.28
Public sector employee	98/99 (99.0%)	1.22	1.16-1.27	84/84 (100.0%)	NA	NA-NA
Private sector employee	297/311 (95.5%)	1.17	1.12-1.23	240/245 (98.0%)	1.20	1.14-1.26
**Father’s occupation**						
Unemployed	167/207 (80.7%)			118/160 (73.8%)		
Worker	204/266 (76.7%)	0.95	0.87-1.04	163/201 (81.1%)	1.10	0.98-1.23
Public sector employee	114/125 (91.2%)	1.13	1.04-1.23	99/99 (100.0%)	NA	NA-NA
Private sector employee	426/436 (97.7%)	1.21	1.13-1.30	342/359 (95.3%)	1.29	1.17-1.42

Multivariate logistic regression analyses were performed to adjust for confounders that may affect the association between risk factors and immunization coverage. After all variables were adjusted, parents’ age, occupational and educational statuses were independently associated with immunization coverage at 6 and 12 months (Table 5). Presence of more than 3 children in the family was also found to be an independent predictive factor of the infant’s immunisation status.

**Table 5:** **Multivariate analysis on predictive factors for vaccination coverage**

	6 months		12 months	
Risk Factor	Adjusted OR (95%CI)	p - value	Adjusted OR (95%CI)	p - value
>3 children	0.36 (0.21–0.63)	< 0.001	0.42 (0.24 – 0.72)	< 0.001
Mother’s age	NA	NA	4.71(2.15 – 10.33)	<0.001
Father’s age	2.76 (1.69 - 4.5)	< 0.001	NA	NA
Mother unemployed	0.14 (0.03 - 0.6)	< 0.001	3.50 (1.35 – 9.12)	< 0.001
Father working in private sector	4.48 (2.24 – 8.95)	<0.001	1.7 (0.91 – 3.17)	< 0.001
Mother’s education	3.56 (19.5 – 6.49)	< 0.001	1.93 (1.18 -3.15)	< 0.001
Father’s education	NA	NA	4.19 (2.51 – 7.00)	< 0.001

## Discussion

Vaccination coverage of infants aged 2-24 months old living in metropolitan Athens visiting tertiary public outpatient care clinic for either acute or routine health visit was evaluated. Although vaccination coverage for DTaP-IPV-Hib and MMR was high, vaccination against pneumococcal, meningococcal infection and hepatitis B is either delayed or incomplete. Risk factors associated with either incomplete or delayed vaccination were explored.

The selection of the herein criteria used in order to define complete vaccination at 6 and 12 months of age were based on most common practices adopted by Greek pediatricians. In Greece, as in most other Mediterranean countries, children’s primary care is anthropocentric and provided by private pediatricians. Overall, parents appear to have a trusting relationship with their private paediatrician [11]. Moreover, during the past few years, since the emergence of economic crisis in Greece, there have been discussions among pediatricians concerning potential need of vaccination priorities reassessment. These refer to vaccinating against pneumococcal disease using the 2 + 1 schedule instead of the 3 + 1 as currently recommended and postponing meningococcal vaccination for after the first birthday since the incidence of meningococcal C infection has significantly decreased post universal vaccination implementation. Although these practices have been publicly discussed among experts, there has been no official change in the NIP. Our “acceptance” of these unofficial changes of the NIP may be criticized. This was decided based on observed practices as reported by other studies [2, 12–14]. It became evident, that if the analyses were performed with more stringent criteria as advised by the NIP, the reported vaccination coverage would be extremely low impeding the identification of factors associated with incomplete vaccination [12, 13].

This may well explain why 40.5% of 6 month old infants had been vaccinated with one dose of MenC vaccine and 43% of 12 month old children with three PCV doses. Nevertheless, when compared to the results of the National vaccination study among children born in 2005 results indicate a significant increase in vaccination coverage for MenC (18% had received two doses at 12 months of age) and PCV (6% had received 3 doses at 12 months of age), possibly because the children included in our cohort were born at least two years later [2]. Although one can postulate that study population recruited in an outpatient department might have introduced a sample bias, a retrospective study among toddlers attending day care in Athens and Viotia (2010-2011), reported vaccination coverage with 3 PCV doses during their first year at 46.8% [13].

In regards to hepatitis B, 56.3% and 77% of children had received three hepatitis B doses by the age of 12 and 18 months respectively. Importantly, possibly because Greek pediatricians recognize the increased risk of horizontal transmission of hepatitis B, minority children had vaccination coverage rates similar to those of non-minority children, in contrast to our findings on other routine vaccines. Moreover, this occurred despite the significantly reduced use of hexavalent vaccine among minority children. Although all vaccines included in NIP are provided free of charge, the hexavalent vaccine was not included in the repertoire of reimbursed vaccines of a major insurance fund at the time of the study. Thus, infants vaccinated with an hexavalent vaccine might significantly differ from other children in reference to their socioeconomic status, parental attitudes to vaccination. It has been well described that Greek It has been well described that Greek pediatricians often prefer to defer initiation of vaccination against hepatitis B until the second semester of life [12]. In a retrospective study among toddlers attending day care centers in Athens (2010-2011), although 94.3% were appropriately vaccinated at the age of 4 years, less than a third (27.7%) had received three hepatitis B vaccine doses during the first year [14]. These results are in accordance with the National vaccination coverage study where although only 36% of infants had received 3 hepB doses at the age of 12 months, the overall vaccination coverage with 3 doses of hepB among 6 years old children was 98%. The authors commented that vaccination coverage was lowest in children living in the Athens Metropolitan area [2]. In our study, the median age of receipt of the first hepatitis B vaccine dose was 173 days and only 43% of infants initiated hepatitis B vaccination before the age of 3 months. Other studies from EU countries also report incomplete vaccination against hepatitis B among their children [15]. Hepatitis B birth dose, which is associated with increased likelihood of early completion of the hepatitis B vaccination is not given in Greece [16]. However, data from Italy where the hexavalent vaccine is used for the vaccination in infants denote significant higher vaccination coverage rates [17]. In concordance to this data, in our cohort, children vaccinated with the hexavalent vaccine were significantly more likely to have received 1 and 3 hepatitis B doses by the age of 6 and 18 months (Figure 1). However, as aforementioned, other confounders such as parental attitudes and economic status may explain the observed difference.

In Greece MMR is administered between 12-15 months of age. In this cohort, 80% of children over 15 months of age had received MMR vaccination by the age of 17 months (within 2 months from NIP recommendations). Although one may postulate that immunization coverage is lower than expected due to the small number of children >18 months included in our cohort (21%) this is unlikely since 95% CIs not significantly wider as shown in Table 3. Vaccination coverage was lower than that reported in either the National aforementioned study (91%) or in the day care center study in Athens (90.5%) [2, 14]. However, in both studies vaccination coverage was estimated at an older age. More importantly these two earlier studies recruited children in schools while our study involved children visiting outpatient clinics. Among the most recently introduced into the NIP programme vaccines (varicella, hepatitis A and rotavirus), varicella vaccination coverage was highest (87.5%, Table 3). Results were similar to those reported from the National study but significantly higher than those described in the day care center study (61%) [2, 14]. This might be due to the recent introduction of the vaccine in the NIP (2006). However, median age of children attending day care study was 4 years and thus vaccination catch up could have been provided. Vaccination coverage for hepatitis A was lower from the National study where 82% of the 6 year old schoolchildren had received two doses but this was possibly due to the different age groups in the two cohorts (Table 3). Finally, the low vaccination coverage against rotavirus was expected since although the vaccine is included in the NIP, it is not fully reimbursed and parents still have to pay out of their pockets for 25% of the vaccine cost (similar for both vaccines available ~28€ for complete vaccination). Moreover, this vaccine unlike most other vaccines, is characterized by the NIP-2011 as "recommended” and not as “necessary” which might additionally explain the low vaccination coverage rates observed.

The proportion of parents not bringing their child’s immunization card along to the outpatient department was small and this may be due to the common practice of reviewing vaccination history in outpatient visits. Characteristics of those parents are easily explainable since they include highly educated Greek parents, most of which have a private paediatrician, which came in for an emergency visit rather than routine care. Also, in our cohort, 9.7% of children were Roma and of those only a third brought their vaccination booklet. This is an overrepresentation of this minority population which consists about 1.5% of Greek population according to data from the Hellenic Ministry of Labour and Social Security. However, in the most recent National vaccination coverage study, researchers included a sub-study of 218 Roma children (median age of 4 years), and found that 87% owned an immunization card [12].

Univariate analysis indicated that immunization coverage was lowest among infants born to young, illiterate and unemployed parents. Moreover, having more than two siblings or belonging to a minority group was also associated with lower vaccination coverage at 6 or 12 months of age (Table 4). Multivariate analysis (Table 5) confirmed that parent’s age, educational and occupational status as well as family size were predictive factors for the infant’s immunization status. Interestingly, belonging to a minority group was not associated with incomplete vaccination coverage during multivariate analysis, which could possibly be explained by the composition of the study population where almost 50% of children were born to immigrants. This might have introduced different confounding factors. As previously suggested, socioeconomic factors such as parental status and family size appear to be the most important predictors associated with under vaccination among Greek children [12]. Interestingly however, in a recent study, higher rather than lower maternal education was associated with incomplete vaccination [14]. Our study confirmed that parental beliefs opposing vaccination is not an important factor in Greece today [12]. These findings may be due to the anthropocentric nature of pediatric care in Greece. More importantly, the composition of our population might also justify the absence of anti-vaccine philosophy since our population consisted of urban working class parents with an over-representation of working immigrants [18]. In contrary to findings from the National study, in this highly urban population distance to the immunization center or private paediatrician was not perceived as a reason to postpone vaccination [12].

This study was performed at the beginning of the economic crisis in Greece. Only, 6.4% of parents reported that they had lost their insurance. Notably, rates of missing opportunity to vaccinate were not higher in this group. It will be important to re-examine this growing proportion of our population today since probably under vaccination among these groups may have increased. Although multiple NGOs have organized actions to accommodate preventive measures such as infant vaccination among Greeks that have recently lost their insurance due to unemployment, these actions were not organised centrally at the time. Previous experience in areas undergoing financial crisis indeed has indicated that children are at risk of experiencing negative health-related consequences [8]. Moreover, it becomes evident that austerity measures across Europe have resulted in decreased access to health care and preventive medicine services affecting mainly children and migrants [19–22].

This study has important limitations. Most importantly, it did not include a representative sample but rather a sample of convenience, namely children visiting the outpatient department of an inner city tertiary care children’s hospital. It has therefore described the vaccination coverage of children of middle to lower socioeconomic status families residing in Attica with an overrepresentation of minority children and under-representation of higher socioeconomic status families or those residing in rural Greece. However, this cohort of children includes those expected to be mostly affected by the ongoing economic crisis [22]. Therefore identifying gaps and risk factors associated with incomplete vaccination coverage during the early years of economic recession will support focusing future public health interventions aiming to improve immunization delivery in Greece. Another significant limitation involves our selecting less stringent criteria to define complete vaccination at 6 and 12 months of age, based on most common practices adopted by Greek pediatricians. This was however, necessary to make useful analyses. Further discussions with pediatricians on potential need for changes of the NIP due to the crisis is important since primary care is provided through an anthropocentric health care delivery system where vaccinations are mandatory without penalty existing for non-compliance.

## Conclusions

This study indicates that at the beginning of the economic crisis in Greece, incomplete vaccination coverage in toddlers is associated with socioeconomic factors such as parental age, educational and occupational status. Since ongoing recession might further affect vaccination coverage, especially among identified groups, the need for further discussions between primary care pediatricians and public health officials, possibly reassessing vaccination priorities under the current economic situation is apparent.

## Abbreviations

BCG: Bacillus Calmette–Guérin vaccine
DTaP: Diphteria, tetanus and acellular pertussis vaccine
EPI: Expanded Program of Immunization
HAV: Hepatitis A vaccine
HepB: Hepatitis B vaccination
Hib: Conjugated haemophilus influenzae type B vaccine
IPV: Inactivated polio vaccine
MCC: Meningococcal serotype C conjugated vaccine
MMR: Measles, mumps and rubella vaccine
NIP: National Immunization Programme
OR: Odds ratio
Ref: Reference (value)
TB: Tuberculosis
VPD: Vaccine preventable diseases
WHO: World Health Organization.
